# Public and patient involvement in quantitative health research: A statistical perspective

**DOI:** 10.1111/hex.12800

**Published:** 2018-06-19

**Authors:** Ailish Hannigan

**Affiliations:** ^1^ Public and Patient Involvement Research Unit Graduate Entry Medical School University of Limerick Limerick Ireland; ^2^ Health Research Institute University of Limerick Limerick Ireland

**Keywords:** clinical trial, cohort studies, participatory, public and patient involvement, quantitative, statistics

## Abstract

**Background:**

The majority of studies included in recent reviews of impact for public and patient involvement (PPI) in health research had a qualitative design. PPI in solely quantitative designs is underexplored, particularly its impact on statistical analysis. Statisticians in practice have a long history of working in both consultative (indirect) and collaborative (direct) roles in health research, yet their perspective on PPI in quantitative health research has never been explicitly examined.

**Objective:**

To explore the potential and challenges of PPI from a statistical perspective at distinct stages of quantitative research, that is sampling, measurement and statistical analysis, distinguishing between indirect and direct PPI.

**Conclusions:**

Statistical analysis is underpinned by having a representative sample, and a collaborative or direct approach to PPI may help achieve that by supporting access to and increasing participation of under‐represented groups in the population. Acknowledging and valuing the role of lay knowledge of the context in statistical analysis and in deciding what variables to measure may support collective learning and advance scientific understanding, as evidenced by the use of participatory modelling in other disciplines. A recurring issue for quantitative researchers, which reflects quantitative sampling methods, is the selection and required number of PPI contributors, and this requires further methodological development. Direct approaches to PPI in quantitative health research may potentially increase its impact, but the facilitation and partnership skills required may require further training for all stakeholders, including statisticians.

## 1 BACKGROUND

Public and patient involvement (PPI) in health research has been defined as research being carried out “with” or “by” members of the public rather than “to,” “about” or “for” them.^1^ PPI covers a diverse range of approaches from “one off” information gathering to sustained partnerships. Tritter's conceptual framework for PPI distinguished between *indirect* involvement where information is gathered from patients and the public, but they do not have the power to make final decisions and *direct* involvement where patients and the public take part in the decision‐making.^2^

A bibliometric review of the literature reported strong growth in the number of published empirical health research studies with public involvement.^3^ In a systematic review of the impact of PPI on health and social care research, Brett et al^4^ reported positive impacts at all stages of research from planning and undertaking the study to analysis, dissemination and implementation. The design of the majority of empirical research studies included in both reviews was qualitative (70% of studies in Brett. et al^4^ and 73% in Boote et al^3^). More significant tensions have been reported in community‐academic partnerships that use quantitative methods rather than solely qualitative methods, for example tensions with the community about having and recruiting to a “no intervention” comparison group.^5^ Particular challenges for PPI have been reported in the most structured and regulated of quantitative designs, that is a randomized controlled trial (RCT), where there is little opportunity for flexibility once the trial has started^6^ and Boote et al^3^ concluded that researchers may find it easier to involve the public in qualitative rather than quantitative research.

If the full potential of PPI for health research is to be realized, its potential and challenges in quantitative research require more exploration, particularly the features of quantitative research which are different from qualitative research, for example, sampling, measurement and statistical analysis. Statisticians in practice have a long history of working with a variety of stakeholders in health research and have examined the difference between an indirect or consulting role for the statistician and a more direct, collaborative role,^7^ yet their perspective has never been explicitly explored in health research with PPI. The objective of this study therefore was to critically reflect on the potential and challenges for PPI at distinct stages of quantitative research from a statistical perspective, distinguishing between *direct* and *indirect* approaches to PPI.^2^

## 2 SAMPLE SIZE AND SELECTION

Quantitative research usually aims to provide precise, unbiased estimates of parameters of interest for the *entire* population which requires a large, randomly selected sample. Brett et al^4^ reported a positive impact of PPI on recruitment in studies, but the representativeness of the sample is as important in quantitative research as sample size. Studies have shown that even when accrual targets have been met, the sample may not be fully representative of the population of interest. In cancer clinical trials, for example, those with health insurance and from higher socio‐economic backgrounds can be over‐represented, while older patients, ethnic minorities and so‐called hard‐to‐reach groups (often with higher cancer mortality rates) are under‐represented.^8^ This limits the ability to generalize the results of the trials to all those with cancer. There is evidence that a *direct* approach to PPI with sustained partnerships between community leaders, primary care providers and clinical trial researchers can be effective in increasing awareness and participation of under‐represented groups in cancer clinical trials^9^, ^10^ and therefore help to achieve the goal of a population‐representative sample.

Collecting representative health data for some groups in the population may only be possible with their involvement. Marin et al^11^ reports on the challenges of identifying an appropriate sampling frame for a health survey of Aboriginal adults in Southern Australia. Access to information identifying Aboriginal dwellings was not publically available, making it difficult to randomly select participants for large population household surveys. Trying to overcome this challenge involved reaching agreement on the process of research for Aboriginal adults with their local communities. An 8‐month consultation process was undertaken with representatives from multiple locations including Aboriginal owned lands in one region; however, it was ultimately agreed that it was culturally inappropriate for the research team to survey this region. The study demonstrated the opportunities for PPI in quantitative research with a representative sample of randomly chosen Aboriginal adults (excluding those resident in one region) ultimately achieved but also the challenges for PPI. The *direct* approach to involvement in this study, after a lengthy consultation process, resulted in a decision not to carry out the planned sampling and data collection in one region with implications for generalization of results and overall sample size.

Of course, given the importance of representativeness in quantitative research, there may be particular challenges for statisticians and quantitative researchers in accepting the term patient or public *representative* with some suggesting PPI *contributor* as a more appropriate term.^6^ PPI representative may suggest to a quantitative researcher that an individual patient or member of the public is typical of an often diverse population, yet there is evidence that the opportunities and capacity to be involved as PPI contributors vary by level of education, income, cognitive skills and cultural background.^12^ Dudley et al carried out a qualitative study of the impact of PPI in RCTs with patients and researchers from a cohort of RCTs.^6^ The types of roles of PPI contributors described by researchers involved in the RCTs were grouped into oversight, managerial and responsive roles. Responsive PPI was described as informal and impromptu with researchers approaching multiple “responsive” PPI contributors as difficulties arose, for example advising on patient information sheets and follow‐up of patients. It was reported that contributions from responsive roles may carry more weight with the researchers in RCTs because it allowed access to a more diverse range of contributors who researchers saw as more “representative” of the target population.

## 3 MEASUREMENT

Measurement of quantitative data involves decisions about what to measure, how to measure it and how often to measure it with these decisions typically made by the research team. Without the involvement of patients and the public, however, important outcomes for people living with a condition have been missed or overlooked, for example fatigue for people with rheumatoid arthritis^13^ or the long‐term effects of therapy for children with asthma.^14^

Core outcome sets (COS) are a minimum set of agreed important outcomes to be measured in research on particular illnesses, conditions or treatments to ensure important outcomes are consistently reported and allow the results from multiple studies to be easily combined and compared. Young reported on workshops to explore what principles, methods and strategies that COS developers may need to consider when seeking patient input into the development of a COS.^15^ The importance of distinguishing between an *indirect* role for patients in COS development where patients respond to a consensus survey or a *direct* role where patients are partners in planning, running and disseminating a COS study was highlighted by delegates in the workshops. While all delegates agreed that participation by patients should be meaningful and on an equal footing with other stakeholders, there was considerable uncertainty on how to achieve this, for example how many patients are needed in the COS development process or what proportion of patients relative to other stakeholders should be included. This raises the issue again of the number and selection of PPI contributors for quantitative researchers, and it was concluded that methodological work was needed to understand the COS development process from the perspective of patients and how the process may be improved for them.

Important considerations in longitudinal research are the number and timing of repeated measurements. From a statistical perspective, measurements on the same subject at different times are almost always correlated, with measurements taken close together in time being more highly correlated than measurements taken far apart in time. Unequal spacing of observation times may be more computationally challenging in statistical analysis of repeated measurements and missing data within subjects over time can be particularly challenging depending on the amount, cause and pattern of missing data.^16^ There are therefore important statistical considerations to be taken into account in the design of longitudinal studies but these have to be balanced with input from PPI contributors on appropriate timing and frequency of data collection for potential participants.

Lucas et al reported on how European birth cohorts are engaging and consulting with young birth cohort members.^17^ Of the 84 individual cohorts identified, only eight had a mechanism for consulting with parents and three a mechanism for consulting with young people themselves (usually “one off” consultations). Very varied follow‐up rates were reported from 13% to 84% more than 10 years after enrolment for individual data rounds of the birth cohorts.^17^ Being motivated to continue to participate may be influenced by whether a participant believes the study is interesting, important, or relevant to them.^18^ One of the key strategies for retention in the Australian Aboriginal Birth Cohort study was partnerships with community members with local knowledge who were involved in all phases of the follow‐up.^19^ Retention rates of 86% at 11‐year follow‐up and 72% at 18‐year follow‐up were reported which demonstrates the potential of a *direct* approach to PPI. Ethical approval for the study involved an Aboriginal Ethical Sub‐committee which had the power of veto and a staged consent was used where participants had the right to refuse individual procedures at each wave. As with all missing data, this has implications for the statistical analysis yet only 10% of participants in this study chose to opt out of different assessments at follow‐up.

### 3.1 Statistical analysis

A report on the impact of PPI found that it had a positive impact at all stages of qualitative research including data analysis but that there was little evidence of its impact on quantitative data analysis.^20^ It was concluded this lack of evidence may reflect a lack of involvement rather than an evidence gap. Booth et al^3^ also suggested that the public may be more comfortable with interpreting interview and focus group data compared with numeric data. Low levels of numerical and statistical literacy in the general population may contribute to this.

Statistical analysis involves describing the data using appropriate graphical and numerical summaries (descriptive statistics) and using more advanced statistical methods to draw inferences about the population using the data from a sample (statistical inference). Choosing appropriate methods for statistical inference, testing the underlying assumptions and checking the adequacy of the models produced requires advanced statistical training and implementing them typically involves the use of statistical software or programming. Statisticians bring this expertise to quantitative health research and while it is important that the chosen methods are adequately communicated to all stakeholders, replicating this type of expertise in PPI contributors seems like an inefficient use of resources for PPI.

Quantitative data are, however, “not just numbers, they are numbers with a context”^21^ and most practising statisticians agree that knowledge of the context is needed to carry out even a purely technical role effectively.^22^ While many associate statistical analysis with objectivity, in practice, statisticians routinely use “subjective” external information to guide, for example the decision on what is a meaningful effect size; whether an outlier is an error in data entry or represents an unusual but meaningful observation; and potential issues with measurement of variables and confounding.^23^ Gelman and Hennin argue that we should move beyond the discussion of objectivity and subjectivity in statistics and “replace each of them with broader collections of attributes, with objectivity replaced by transparency, consensus, impartiality and correspondence to observable reality, and subjectivity replaced by awareness of multiple perspectives and context dependence.”^23^ This debate within statistics is relevant for PPI where the perceived objectivity and standardization of statistical analysis can be used as a reason for lack of involvement.

External information and context are particularly important in statistical modelling where statisticians are often faced with many potential predictors of an outcome. The “best” way of selecting a multivariable model is still unresolved from a statistical perspective, and it is generally agreed that subject matter knowledge, when available, should guide model building.^24^ Even when the potential predictors are known, understanding the causal pathways of exposure on an outcome is challenging where the effect of a variable on the outcome can be direct or indirect. Christiaens et al^25^ used a causal diagram to visualize the relationship between pain acceptance and personal control of women in labour and the use of pain medication during labour. Their analysis accounted for the maternal care context of the country where the women were giving birth and other characteristics such as age of the woman and duration of labour. The choice of these characteristics was underpinned by a literature review but women who have given birth also have expert knowledge on why they use pain relief and how other variables such as their personal beliefs and social context might influence that decision.^26^

Collaborative or participatory modelling is an approach to scientific modelling in areas such as natural resource management which involves all stakeholders in the model building process. Participants can suggest characteristics for inclusion in the model and how they may impact on the outcome. Causal diagrams are then used to create a shared view across stakeholders.^27^ Rockman et al^28^ concluded, in the context of marine policy, that “participatory modelling has the potential to facilitate and structure discussions between scientists and stakeholders about uncertainties and the quality of the knowledge base. It can also contribute to collective learning, increase legitimacy and advance scientific understanding.”

There is emerging evidence that the importance of PPI in the development and application of modelling in health research is being recognized. Van Voorn^29^ discussed the benefits and risks of PPI in health economic modelling of cost‐effectiveness of new drugs and treatment strategies, with public and patients described as the missing stakeholder group in the modelling process. The potential benefits included the expertise that patients could bring to the process, a greater understanding and possible acceptance by patients of the results of the models and improved model validation. The risks included potential patient bias and the increased resources required for training. The number and selection of patients to contribute to the process was also discussed with a suggestion to include patients “who were able to take a neutral view” and “at least five patients that differ significantly in their background,” again highlighting the focus of quantitative researchers on bias and sample size. The role for this type of participatory modelling in informing debate on public health problems is increasingly being recognized, drawing on the experience of its use in other areas where optimal use of limited resources is required to address complex problems in society.^30^

## 4 CONCLUSIONS

Statistical analysis of quantitative data is underpinned by having a representative sample, and there is evidence that a *direct* approach to PPI can help achieve that by supporting access to and increasing participation of under‐represented groups in the population. The *direct* approach has also demonstrated its potential in the retention of those recruited over time, thus reducing bias caused by missing data in longitudinal studies. At all stages of statistical analysis, a statistician continuously refers back to the context of the data collected.^22^ Lay knowledge of PPI contributors has an important role in providing this context, and there is evidence from other disciplines of the benefits of including this knowledge in analysis to support collective learning and advance scientific understanding.

The *direct* approach to PPI where patients and the public have the power to make decisions also brings challenges and the statistician needs to be able to clearly communicate the impact of each decision on the scientific rigour and validity of sampling, measurement and analysis to all stakeholders. Decisions made on participation impact on generalizability. Participatory modelling requires facilitation and partnership skills which may require further training for all stakeholders, including statisticians.

The *direct* and *indirect* role for PPI contributors mirrors what happens for statisticians in practice. Statisticians can have a consultative role, that is answering a specific statistical question or a collaborative role where a statistician works with others as equal partners to create new knowledge, with professional organizations for statisticians providing guidance and mentorship on moving from consulting to collaboration to leadership roles.^7^, ^31^ Statisticians therefore bring very relevant experience and understanding for PPI contributors on the ladder of participation in health research. Further exploration is required on the impact of *direct* compared to *indirect* involvement in quantitative research, drawing on the evidence base for community‐based participatory research in quantitative designs^9^ and the framework for participatory health research and epidemiology.^32^, ^33^

## CONFLICT OF INTERESTS

No conflict of interests.
